# Therapeutic Opportunities through the Modulation of Endocannabinoid Transport

**DOI:** 10.4172/2167-7689.1000e123

**Published:** 2013-12-10

**Authors:** Jason J Guo, Mark K Williams, Alexandros Makriyannis

**Affiliations:** Center for Drug Discovery, Northeastern University, 360 Huntington Avenue, Boston, MA 02115, USA

The endocannabinoid system is comprised of cannabinoid receptors (CB1 and CB2), a group of endogenous neuromodulatory lipids (endocannabinoids), and the machinery for their biosynthesis, metabolism, and transit that are involved in a variety of physiological processes including pain, appetite, memory, inflammatory and immune responses. In the brain, endocannabinoids are primarily involved in retrograde signaling, being synthesized and released from postsynaptic neurons to stimulate CB1 receptors located on the presynaptic neurons. Here, we provide an overview of the current research on the transport of endocannabinoids across plasma membranes and their intracellular trafficking with an emphasis on various potential targets for developing therapeutic drugs.

## The Endocannabinoid System

The two most extensively studied endocannabinoids are anandamide (AEA or N-arachidonylethanolamine) and 2-arachidonylglycerol (2-AG). They are believed to be synthesized “on-demand” in response to various physiological stimuli to modulate intracellular secondary messengers upon receptor activation. Imbalances in the endocannabinoid system, either in the central nervous system (CNS) or peripheral tissues, is associated with many types of pathologies. In particular, changes in tissue concentrations of anandamide and 2–arachidonoylglycerol have been observed in pain, inflammation, obesity, neurological and immunological disorders.

## Endocannabinoid Transport

During neurotransmission, stringent regulation of the receptor mediated signaling is required along with rapid removal of the endocannabinoids from the synaptic cleft. A growing body of evidence has shown that endocannabinoids can rapidly cross the plasma membrane followed by carrier-mediated transport to their intracellular sites of sequestration or hydrolysis in different subcellular locations [1,2]. Several mechanisms, including passive diffusion, facilitated diffusion, membrane transporter, and/or endocytosis, have been proposed to account for the rapid translocation of endocannabinoids across the plasma membrane. A number of intracellular carrier proteins have also been implicated to be able to shuttle the endocannabinoids from the plasma membrane to the subcellular sites for enzymatic inactivation. Even though controversies remain, we have come a long way towards the understanding of the endocannabinoid transport process and it is now recognized that different tissues/cell types are more likely to utilize one or a combination of several different mechanism(s).

For many years it was understood that transport of lipid molecules such as long-chain fatty acids through the cell membrane occurred by passive diffusion. However, a fundamental shift in understanding has occurred and now it is generally accepted that fatty-acids cross the cell membrane by a protein-mediated mechanism involving either specific transporter(s) and/or carrier protein(s). Pharmacological studies [3] indicated that the uptake of anandamide can be inhibited by select fatty acid amide derivatives such as N-(4-hydroxyphenyl)-arachidonamide (AM404) in a dose dependant manner, suggesting there is a putative membrane transporter involved in this process. Additionally, these compounds do not produce observable cannabis-like effects in drug discrimination tests. This raises the possibility that targeting the transport process may provide a range of novel potential targets that can modulate AEA-related signaling to treat pain, addiction and other disorders.

The structural similarity of endocannabinoids to fatty acids suggests that they may share similar transport mechanisms. Contemporary models based on the characterization of “fatty-acid transporters” are comprised of, but not limited to, plasma membrane-associated fatty-acid binding proteins (FABPs), the cytosolic and circulating FABPs as well as a family of fatty-acid transport proteins (FATP 1–6). These intracellular carrier proteins can provide potential targets for the development of therapeutics that modulate lipid-signaling pathways. Currently, the functions of individual components of the fatty-acid transporter model are being elucidated, and intracellular carrier proteins that shuttle the endocannabinoid anandamide from the plasma membrane to its intracellular targets have been identified. These include fatty acid binding proteins, albumin, heat shock protein 70, and the fatty acid amide hydrolase-like anandamide transporter protein (FLAT) [4–6]. It has also been reported that circulating levels of adipocyte FABP (FABP4) serves as an indicator for lipid metabolic dysfunctions. Several independent clinical studies measuring circulating levels of FABP4, have demonstrated that an elevation in circulating FABP4 and an increase in serum lipids, inflammatory markers MCP-1 and hsCRP are strongly associated in patients with metabolic dysregulations such as atherosclerosis, type 2 diabetes and dyslipidemias. FABP4 likely represents an important pathophysiological mediator of metabolic aberrations exerting a dramatic impact on obesity, insulin resistance, type 2 diabetes, fatty liver disease and atherosclerosis, all of which are conditions associated with the endocannabinoid system.

## Potential Therapeutic Applications for Inhibitors of Endocannabinoid Cellular Uptake

A number of compounds that inhibit the cellular uptake of anandamide have been developed; however, in most cases the mechanism by which they inhibit cellular uptake has yet to be fully determined. Even so, it has been demonstrated that AM404, AM1172, VDM11 and UCM707 as well as OMDM1, OMDM2 and LY2183240, can significantly reduce the cellular uptake of anandamide. Moreover, one or more of these compounds have been found to decrease carcinoma cell proliferation and tumor growth, and are effective in animal models against indicators of inflammation, pain, multiple sclerosis, colitis and anxiety, diarrhea. In addition, both AM404 and VDM11 were found to inhibit the reinstatement of nicotine-seeking behavior in rats, and that AM404 also prevents the development of nicotine dependence in rats. Evidence has also been obtained, from murine models, that AM404 may be effective against obsessive-compulsive disorder. Though AM404 has off-target interactions that inhibit COX-1, COX-2 and prostaglandin synthesis, this compound has been found to reduce signs of visceral pain in mice in a manner that appears to be CB1 receptor-mediated and is a molecule with overall interesting therapeutic effects that make it a candidate for further studies. It has also been reported that SB-Fl-26, an inhibitor of the intracellular fatty acid binding proteins, displays antinociceptive activity in a mouse model of inflammatory pain. Continuing effort in the design and development of pharmacological agents that inhibit anandamide uptake can serve to control lipid-signaling pathways, inflammatory responses and metabolic dysfunction, offering a new class of multi-indication pharmacophore with high potential for future therapeutic applications in the prevention and treatment of metabolic associated disease.
